# Exploring the associations of weather and climate with HIV in sub-Saharan Africa: a systematic review

**DOI:** 10.1136/bmjph-2024-001805

**Published:** 2025-08-18

**Authors:** Matylda Buczkowska, Adam Trickey, Gina E C Charnley, Anthea Gabot, George Hutchings, Collins C Iwuji, Ilan Kelman

**Affiliations:** 1 Institute for Global Health, University College London, London, UK; 2 Bristol Medical School, University of Bristol Population Health Sciences, Bristol, UK; 3 Global Health Resilience Group, Barcelona Supercomputing Center—Centro Nacional de Supercomputación, Barcelona, Spain; 4 Department of Infectious Disease Epidemiology, Imperial College London, London, UK; 5 Department of Global Health and Infection, Brighton and Sussex Medical School, University of Sussex, Brighton, UK; 6 Population Science, Africa Health Research Institute, Durban, South Africa; 7 Institute of Global Health and Institute for Risk and Disaster Reduction, UCL, London, UK; 8 University of Agder, Kristiansand, Norway

**Keywords:** HIV, Systematic Review, Environmental Exposure

## Abstract

**Background:**

Joint United Nations Programme on HIV/AIDS has previously hypothesised that in sub-Saharan Africa, extreme weather/climate and HIV might be associated. A systematic review was conducted to summarise current evidence on the indirect associations between weather/climate variability and HIV-related measures (such as risk behaviours and access to care) in sub-Saharan Africa. This review does not assess environmental mediation of viral transmission.

**Methods:**

Five literature databases (Web of Science, PubMed, SCOPUS, EMBASE and Global Health) were searched for relevant qualitative and quantitative studies that contained data on associations between weather/climate variables (including extreme weather events and changes in precipitation and temperature) and HIV measures (including HIV risk behaviours and measures of HIV transmission and progression) in the general population of sub-Saharan Africa up to 6 April 2024. Results were summarised through narrative synthesis.

**Results:**

Overall, 5853 non-duplicate papers were retrieved for abstract screening, with 57 studies selected for full-text screening. Of those, 20 studies (14 quantitative and 6 qualitative) were included in the review. Most studies suggested that weather/climate variability was associated with worsening of HIV-related outcome measures. Drought was the most frequently reported weather/climate exposure (12 studies in total), while HIV prevalence and antiretroviral therapy uptake were the most frequently reported HIV measures (10 and 9 studies, respectively). Few studies analysed data from longitudinal datasets and research gaps were identified on West and Central Africa, children and key populations such as female sex workers.

**Conclusions:**

Despite potential associations between weather/climate variability and HIV measures, primarily between droughts and HIV prevalence, there has been limited research published on the topic. The current evidence base is sparse, heterogeneous and insufficient to establish causality. The review highlighted the need for using longitudinal datasets to assess directionality and mediators of weather/climate-HIV relationships, while data on West and Central Africa, children and key populations should be incorporated in future research.

**WHAT IS ALREADY KNOWN ON THIS TOPIC**

- Few studies have investigated the relationship between weather/climate exposures and HIV measures in sub-Saharan Africa.

**WHAT THIS STUDY ADDS**

- The results of the systematic review showed that the association between droughts and increased risk of HIV prevalence was the most frequently reported association in quantitative studies, while the association between drought and decreased antiretroviral therapy use was the most frequently reported association in qualitative studies. Drought was also associated with increased HIV risk behaviours, such as condomless sex. Women in rural areas and younger individuals were overall more affected in the studies, which showed an association between extreme weather/climate and HIV measures than urban, male and adult individuals.

**HOW THIS STUDY MIGHT AFFECT RESEARCH, PRACTICE OR POLICY**

- Individuals could be differentially affected by weather/climate variability due to intersecting vulnerabilities, identifying these groups can inform interventions to protect the health of people living with HIV and reduce the risk of acquisition in those who are HIV negative.

## Introduction

 Shifts to the climate and the Earth’s atmosphere are changing the frequency, intensity and duration of weather extremes, such as floods, storms and droughts,^1^ which can influence physical and mental pathologies in humans.^2^ For example, intense heat may aggravate cardiovascular and respiratory diseases, while heavy rainfall can affect the distribution of vector-borne diseases.^3^ Droughts and flooding may impact crop production and food security, affecting malnutrition, migration and access to healthcare.^3^ In sub-Saharan Africa (SSA), changes to climate variability, low adaptive capacity and a high burden of infectious diseases contribute to the region’s vulnerability.^3^ Research investigating associations between weather/climate variability and health in SSA continues to identify new potential effects, such as outcomes relating to HIV.^4^ In 2022, 65% of people living with HIV (PLHIV) resided in SSA and 60% of all AIDS-related deaths in 2022 occurred in that region, approximately 400 000 in total.^5^ Although there is currently no cure for HIV, antiretroviral therapy (ART) suppresses an individual’s viral load, halts disease progression and vastly reduces the risk of transmission.^6^ Of the estimated 25.6 million PLHIV in the African region, only 81% were on ART at the end of 2022.^4^

The relationship between weather/climate exposures and HIV is not direct but rather operates through complex, interconnected pathways. Theoretical frameworks linking environment to HIV outcomes^7 8^ suggest that events such as droughts or floods can influence HIV-related risks through mechanisms like disrupted livelihoods, migration and barriers to healthcare access. Despite these plausible pathways, no review to date has comprehensively synthesised this growing but disparate literature with a specific focus on SSA. However, because of increasing temperatures and other changing weather statistics in SSA, additional research is required to determine the extent to which weather/climate could affect HIV risks.^4^ A systematic review was conducted to comprehensively appraise and summarise current evidence on the associations between weather/climate exposures and HIV-related measures in SSA, to identify research gaps in the topic area and to facilitate future work in the field. This review focuses on how weather/climate may act as a structural or contextual determinant of HIV outcomes, rather than implying a direct biological effect on transmission.

## Methods

Preferred Reporting Items for Systematic Reviews and Meta-Analyses guidelines were followed when designing this systematic review^9^ (online supplemental table 1).

### Population, weather/climate and HIV measures

The population included in this review was the general human population residing in SSA with or without HIV. The exposures of interest were extreme weather events as well as variables representing weather and climate variabilities. When investigating the exposures, original names of the variables defined by the authors of reviewed papers were retained, despite some of them not being officially accepted by major weather or climate organisations (such as ‘rainfall shock’). Although ‘weather and ‘climate’ have distinct definitions (table 1), the term ‘weather/climate’ is used throughout this review to encompass both. This decision reflects the language used in many of the included studies, which often did not distinguish clearly between the two. Therefore, the combined term allows us to consistently describe a wide range of exposures without introducing artificial separation where none was defined in the source data. The HIV variables included HIV risk behaviours (condomless sex, intergenerational sex, sexual violence, transactional sex, sex with multiple partners), HIV transmission measures (HIV prevalence, HIV incidence, HIV testing) and HIV progression measures (HIV treatment, HIV-1 viral loads, CD4 cell counts, AIDS status and mortality among PLHIV). Definitions of selected measures are provided in table 1. These measures do not refer to changes in viral transmissibility itself, but to changes in population-level burden, risk exposure or healthcare access.

**Table 1:** Definitions of terms utilised in the review

Weather	A state of the atmosphere at a particular time, as defined by the various meteorological elements, including temperature, precipitation, atmospheric pressure wind and humidity.^45^
Climate	‘Average weather’ or more rigorously, a statistical description in terms of the mean and variability of relevant quantities over a period of time ranging from months to thousands or millions of years. The classical period is >30 years, as defined by the World Meteorological Organization (WMO).^45 56^
Climate change	Climate change refers to a change in the state of the climate that can be identified (eg, by using statistical tests) by changes in the mean and/or the variability of its properties and that persists for an extended period, typically decades or longer.^56^
Extreme weather event	Meteorological condition that is rare for a particular place and/or time, such as an intense storm or heat wave. Definitions of ‘rare’ vary, but an extreme weather event would normally be as rare as or rarer than the 10th or 90th percentile of the observed probability density function.^56^
HIV incidence	The estimated number of persons newly acquiring HIV during a specified time period.^57^
HIV prevalence	The number of people with HIV at a given time regardless of the time of infection.^57^
HIV viral suppression	Having less than a certain number of copies of HIV-1 RNA per millilitre of blood, for example, the US Centres for Disease Control and Prevention defines this cut-off at 200.^57^
AIDS	Stage 3 of HIV infection, when the immune system of a person living with HIV becomes severely compromised (measured by CD4 cell count) and/or the person becomes ill with an opportunistic infection.^57^

### Inclusion criteria (Population, Exposure, Comparator, Outcome framework)

- Population: studies involving individuals of any age living in SSA, with or without HIV.
- Exposure: any defined weather or climate variable (drought, flood, temperature variability, heavy rainfall, heatwaves).
- Comparator: not required.
- Outcome: any HIV-related measure, including HIV risk behaviours (such as condomless sex, transactional sex), transmission (such as prevalence, incidence, testing) and progression (such as ART adherence, viral load, mortality).

### Exclusion criteria

- Studies conducted outside SSA.
- Duplicate papers, commentaries, editorials, reviews, theses, letters and conference abstracts.
- Studies lacking a clearly defined weather/climate exposure or a measurable HIV-related outcome.
- Studies using future projections or predictive modelling.
- Studies that used vague descriptions of exposures (such as ‘bad weather’) without reference to a specific variables or metrics.

All searches were not limited by a year of publication or language, although all words used were in English. Both quantitative and qualitative studies were included in the review. We included studies that examined main effects (weather/climate as an exposure and HIV-related measure as the outcome), moderation (weather/climate or HIV measure acting as a moderator in an association between two variables) or mediation (where weather/climate affects an HIV outcome through an intermediate variable).

### Search strategy

Five electronic databases (Web of Science, PubMed, Scopus, Embase and Global Health) were searched for peer-reviewed literature. To identify relevant studies, a comprehensive search strategy was developed, which combined free-text keywords, subject headings (including Medical Subject Headings and Emtree terms) and Boolean operators. The search strategy combined three primary conceptual domains: HIV-related terms, weather/climate-related terms and geographical terms for SSA. Historical names for countries were included to account for older publications, which may refer to countries by their colonial or previous names, particularly relevant given that our search included studies without a time restriction.^10^ Broad and specific weather/climate terms were used to maximise sensitivity, given the lack of consistent terminology in the literature and frequent conflation of ‘climate’ and ‘weather’. Although the review does not focus on climate change as a distinct phenomenon (eg, future climate projections), climate change is associated with changes in intensity, duration, magnitude and frequency of extreme weather events.^1^ Hence, climate change-related terms were included in the search strategy, which often appear interchangeably with weather terms in the literature. Search strategies were adapted to each electronic database through combinations of different search strings to enhance search’s specificity. Research articles published up to 6 April 2024 were systematically retrieved from each database. All search strategies are available inonline supplemental table 2. Reference lists of identified articles and files were checked, and further studies were manually processed, if deemed relevant.

### Study screening

Records retrieved from the search strategies were input into EndNote 20 library,^11^ where duplicate files were removed. All records were later exported to Rayyan web tool,^12^ where two researchers (MB and AG/GH) screened the titles and abstracts of all papers independently, based on inclusion and exclusion criteria. A third researcher resolved the discrepancies (AG/GH). Following that, full-text records were screened in Rayyan by two independent researchers (MB and AG/GH). As before, a third researcher (AG/GH) resolved any discrepancies. Reasons for excluding papers were recorded.

### Data extraction

Data extraction forms were pretested by one author (MB) and approved by all authors. The forms included author details, study aims, study design, time frame, countries, sample characteristics, type of weather/climate event, type of HIV measure, key conclusions (for both quantitative and qualitative studies), datasets used, type of analysis and statistical methods used (quantitative studies), key themes identified and their interpretation (qualitative studies). Data from the included studies were extracted by three authors independently (MB, AG and GH) and later jointly to resolve discrepancies. Where necessary, the corresponding author of included studies was contacted to obtain further information. All the information was recorded and stored in a customised Microsoft Excel spreadsheet.

### Thematic review

The authors utilised a narrative synthesis method to describe the overarching influence of weather/climate variables on HIV measures in SSA. The study aim was answered by evaluating, categorising and concluding the information extracted from the selected papers. Descriptive analysis and tabulations were used to summarise the trends of the relevant selected articles. MapChart application^13^ was used to graphically represent the countries which were studied. Meta-analysis was not performed due to a low number of articles that met the criteria for inclusion and the heterogeneity of the exposures and the outcomes.

### Quality assessment

Quantitative and qualitative quality assessment tools were developed. The quantitative tool was based on ROBINS-E and CASP tools for cohort/cross-sectional data assessment.^14 15^ The tool contained 11 questions, and each paper was given a score from 0 to 11, with a higher score indicating a higher quality of the paper. The qualitative tool was based on CASP and JBI tools for qualitative studies.^16 17^ The tool contained seven questions, and each paper was given a score from 0 to 7, with a higher score indicating a higher quality of paper. Quality assessment tools were pretested by one author (MB) and approved by all authors. Following that, three authors assessed the quality of the studies independently (MB, AG and GH) and later jointly to resolve discrepancies.

## Results

**Figure 1:**
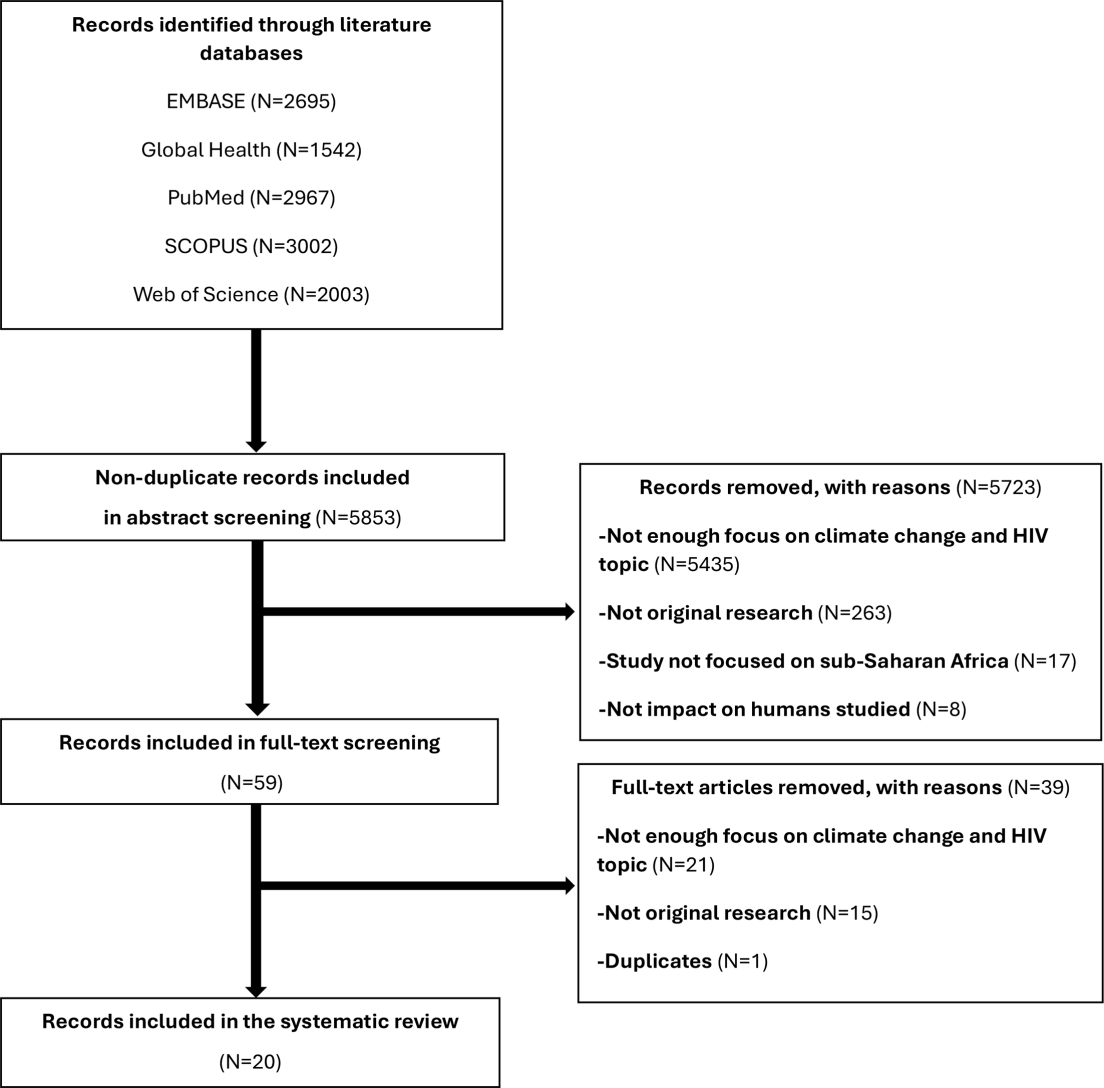
PRISMA flowchart of included studies. PRISMA, Preferred Reporting Items for Systematic Reviews and Meta-Analyses.

Overall, 5853 non-duplicate papers were retrieved for abstract screening, and 57 studies were selected for full-text screening. Out of those, 20 studies (14 quantitative and 6 qualitative) were selected for inclusion in the review (figure 1).

### Quantitative studies

#### Studies characteristics

**Figure 2:**
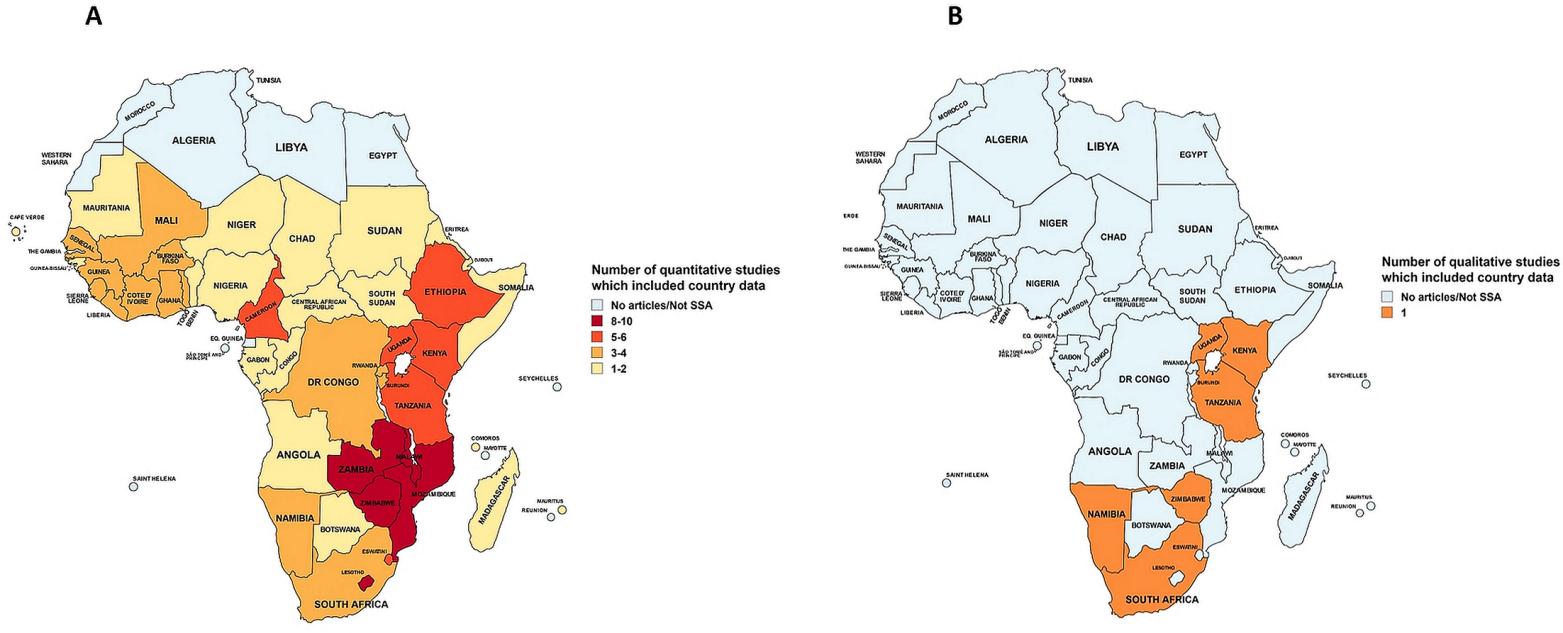
Countries represented in quantitative (**A**) and qualitative studies (**B**). SSA, sub-Saharan Africa.

A summary of the quantitative studies is presented in online supplemental table 3. Fourteen quantitative studies were selected in total.^18^,^31^ There were seven individual-level studies,^22^,^2428^ five of which were cross-sectional,^22 24 28 29 31^ two of which were longitudinal^23 30^ and seven ecological studies.^18^,^2125^ Overall, countries within southern and eastern parts of SSA were studied in the majority of the studies (figure 2A). The countries which were studied the most frequently were Lesotho,^1819 21 22 24^,^26 28 30 31^ Zambia^1819 21 22 25^,^28 30 31^ and Malawi^18^,^2225 26 28^ (figure 2A), while 10 studies included data from more than one country.^18^,^2225 26 28 30 31^

Seven studies had participants who were both men and women.^22^,^2428^ One study focused only on women^18^ and in six studies,^19^,^2125^ the sex of participants was not stated. Ten papers studied only adults (defined as ≥15 years old),^18^,^2224 28^ one studied individuals of any age^23^ and three focused specifically on children below 5 years old.^25^,^27^

The weather/climate exposures studied the most frequently were precipitation changes: drought,^1820 22^,^27 29 31^ ‘rainfall shock’,^21^ heavy rainfall^20 28^ and rainfall variability^30^ (table 2). Two studies also investigated changes in temperature^19 20^: heat waves,^20^ cold snaps^20^ and temperature variability.^19^ The most frequently studied HIV-related measures were HIV prevalence^18^,^2124^ and condom use^1822^,^24^ (table 2).

**Table 2:** Numerical summary of weather/climate exposure-HIV measure association pairs used in the included quantitative studies

		Precipitation changes				Temperature changes		
		Drought (N=10)	Rainfall shock (N=1)	Heavy rainfall (N=2)	Rainfall variability (N=1)	Heat waves (N=1)	Cold snaps (N=1)	Temperature variability (N=1)
HIV risk behaviours	Condom use (N=3)	3	0	0	0	0	0	0
	Transactional sex (N=3)	2	0	0	0	0	0	1
	Forced sex/sexual violence (N=1)	1	0	0	0	0	0	0
	Number of sexual partners (N=3)	2	0	1	0	0	0	0
	Intergenerational sex (N=1)	1	0	0	0	0	0	0
HIV transmission measures	HIV prevalence (N=10)	7	1	2	0	1	1	1
	HIV incidence/recent HIV (N=1)	1	0	0	0	0	0	0
	HIV testing/diagnosis (N=1)	1	0	0	0	0	0	0
HIV progression measures	Gaps in care/ART treatment (N=3)	2	0	0	1	0	0	0
	HIV viral load (N=2)	1	0	0	1	0	0	0
	CD4 cell count (N=1)	1	0	0	1	0	0	0
	Mortality among people with HIV (N=1)	0	0	0	1	0	0	0

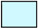
 No studies investigating the association. 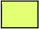 One study investigating the association. 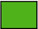 Two or more studies investigating the association.

ART, antiretroviral therapy.

#### Associations between weather/climate and HIV risk behaviours

All seven studies investigating associations between drought and condomless sex,^18 22 24^ transactional sex (primarily in rural females),^24 29^ intergenerational sex^24^ and forced sex^24^ found that drought was correlated with an increased risk of those measures. However, the association between drought and condomless sex was only present in rural females in one study,^24^ while intergenerational and forced sex risk increased only in urban females. Drought was not associated with higher number of sexual partners in one study,^22^ and the association was only present among rural adolescent males in another.^24^ Heavy rainfall was associated with increased risk of a high number of sexual partners,^28^ while warmer temperatures were associated with higher risk of engaging in transactional sex.^19^

#### Associations between weather/climate and HIV transmission

Four main effect/mediation studies on drought and HIV prevalence found that exposure to drought was associated with increased HIV prevalence.^18 20 24 29^ One study found this effect in young rural females only, while drought had a protective effect on HIV prevalence in young males.^24^ ‘Rainfall shock’ was associated with increased HIV prevalence, but only in rural areas.^21^ Heavy rainfall was associated with higher HIV prevalence in two studies,^20 28^ but only among individuals in rural areas and individuals above 30 years old in one of them.^28^ No association was found between ‘cold snaps’, heat waves and HIV prevalence in one study.^20^ However, temperature variability was linked with increased HIV prevalence in another.^19^ In the only study measuring HIV incidence, drought was associated with decreased HIV testing and increased HIV incidence, but only in rural females.^31^

#### Associations between weather/climate and HIV progression measures

Lower rainfall was associated with increased mortality among PLHIV, increased percentage of individuals with unsuppressed HIV viral loads and longer gaps in care in one study.^30^ In the same study, it was also weakly associated with higher CD4 cell counts.^30^ Drought was not associated with the percentage of the population with suppressed viral loads nor ART treatment in one study^30^ but was linked with decreased ART medication possession ratio and retention in care in another.^23^

#### Potential indirect pathways

In many quantitative studies, the underlying mechanisms linking weather/climate and HIV were not explored in depth. Nonetheless, a few studies offered some insights. For example, one study^30^ found that HIV clinics in areas experiencing lower than average rainfall recorded fewer visits from PLHIV. This decline in attendance might be a plausible pathway contributing to increased mortality rates and higher viral loads. Another study^31^ identified associations between drought and poverty (particularly in rural areas) as well as between poverty and transactional sex. These findings suggested that transactional sex, especially among individuals in the lowest wealth quintiles, might have posed an increased risk of HIV transmission following drought conditions. However, the study did not test a formal mediation pathway to establish these links. A structural equation modelling study by Kelly *et al*^18^ demonstrated that the relationship between drought and the proportion of adults living with HIV who were women was mediated by food insecurity. In another study,^24^ temporary migration was strongly associated with HIV among young people, but migration itself was not found to be linked to drought. Similarly, Burke^21^ found no evidence for temporary migration as a significant pathway between rainfall shocks and HIV but suggested that having multiple or casual sexual partners might have been associated with such climatic events. On the other hand, Baker^19^ found that male migration and men paying for sex were linked to higher temperatures, indicating a different climate-related behavioural pathway.

#### Quality assessment

Studies which found a negative association between weather/climate variables and HIV measures had an average score of 8.4, while the studies with conflicting results had an average score of 8.0 (online supplemental table 4). The study, which found no association, had a score of 5.0. Studies which received lower scores^1825^,^27^ tended to have multiple methodological limitations. These included unclear or undefined study populations, inconsistent or absent inclusion/exclusion criteria and lack of adjustment for key confounders. Furthermore, missing data handling was rarely described, and in a few cases, results were not fully aligned with the methods described, which limited the interpretability of their findings.

### Qualitative studies

#### Studies characteristics

A summary of qualitative studies is presented in online supplemental table 5. Most studies used interviews to gather their data,^32^,^36^ while one study took an ethnographic research approach.^37^ Additionally, five of the studies performed thematic analysis^3234^,^37^ and one undertook a rapid assessment.^33^ In total, there were at least 418 participants (the number of participants was not reported in Kandawasvika *et al*). All papers studied one country and focused on analysing data from primarily rural regions (figure 2B). More specifically, data from Namibia, Uganda, Tanzania, South Africa, Zimbabwe and Kenya were included (figure 2B). Drought,^35 37^ flood,^32 36^ heavy rain,^34 37^ heat^34^ and Cyclone Idai^33^ were investigated as the exposures (online supplemental table 6), while ART treatment,^32^,^37^ HIV testing,^32^ transactional sex,^37^ forced sex^32^ and condom use^32^ were studied as the HIV measures (online supplemental table 6).

#### Associations between weather/climate and HIV risk behaviours

**Figure 3:**
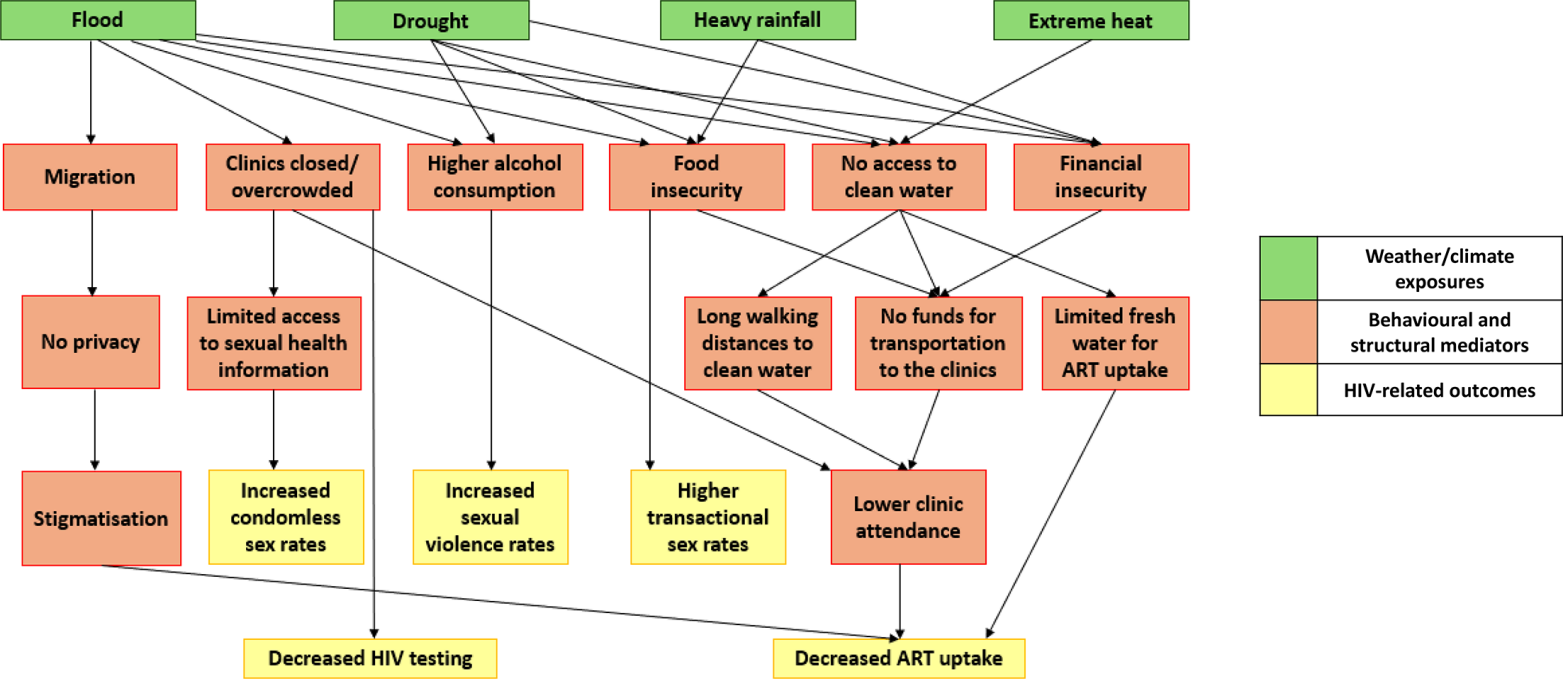
Conceptual pathways between weather/climate and HIV-related outcomes, illustrating indirect behavioural and structural mechanisms identified in qualitative studies. ART, antiretroviral therapy.

Walking long distances to access water following a flood^32^ as well as migration following a flood^32^ were reported to lead to a higher risk of forced sex, especially among women and children. Respondents reported that drought, flood, heavy rainfall and heat all increased food insecurity,^32 34 37^ which made individuals resort to transactional sex to feed their families.^37^ Information on family planning and HIV was limited during a flood, which increased the occurrence of condomless sex.^37^ Higher consumption of alcohol during a flood was also reported to be linked with increased sexual violence (figure 3).^32^

#### Associations between weather/climate and HIV progression measures

One study reported that floods made it difficult for HIV healthcare providers to conduct HIV testing, partially due to increased migration (figure 3).^32^

#### Associations between weather/climate and HIV progression measures

Most studies found that extreme weather/climate events were associated with worse access to ART (figure 3). The only study with conflicting results investigated cyclones as the exposure,^33^ while the remaining studies focused primarily on droughts and extreme rainfall/floods. In this study, the researchers found that following Cyclone Idai, fewer people sought ART, while availability of ART services for PLHIV increased. However, the study only received one point on a 7-point scale in the quality assessment tool; therefore, the results are likely severely biased. Food, water and financial insecurities following a drought, heat, flood or heavy rainfall led to some individuals not being able to attend clinics, for example, due to a lack of transport, which decreased ART uptake.^32 34 35 37^ Relocation to displacement camps following a flood led to stigmatisation and a lack of privacy to take ART.^32^ Scarce water access linked with droughts limited ART intake due to long walking distances to water holes, limited time to go to the clinic and yielded insufficient access to clean water to take the medication.^35^ Floods led to clinics being closed, and a higher volume of patients in the clinics that remained open, which affected HIV testing (figure 3).^32 36^

#### Quality assessment

The average score for studies which found a negative association between weather/climate exposures and HIV measures was 4.6, while for the study which presented conflicting results, the score was 1.0 (online supplemental table 7). Several studies^32 33 36^ received lower scores due to limited detail in their methods and interpretation. Common issues included a lack of a clear description of the study population or recruitment strategy. None of the qualitative studies included a statement on researchers’ positionality. Interpretation of qualitative research can be affected by personal biases, particularly with a topic as highly sensitive as HIV. Therefore, a failure to include an examination of reflexivity could introduce bias into the results.

## Discussion

Most studies included in this systematic review reported that weather/climate variables were associated with worsening of HIV outcome measures. Among studies investigating the links between weather/climate variability and HIV risk behaviours, drought was associated with an increase in condomless sex, transactional sex, intergenerational sex and forced sex in quantitative studies. However, there is a need to investigate other weather/climate exposures in quantitative studies to understand whether similar patterns would emerge. For example, floods and heavy rainfall were also associated with increased frequency of HIV risk behaviours in qualitative studies.

Among studies investigating the correlations between weather/climate variability and HIV transmission measures, drought and warming temperatures were reported to be associated with increased HIV prevalence, HIV incidence and lowered HIV testing in quantitative studies, with the strongest evidence seen among adolescent females. Young women may be vulnerable as economic and social power structures often give them less power to negotiate safe sex practices.^38^ Moreover, some of the studies found negative associations between weather/climate variability and HIV measures only in rural populations. Much of SSA is experiencing high rates of urbanisation, which could potentially lead to an expansion in access to HIV care or overwhelm the capacity to deliver services.^39^

Regarding HIV progression measures, lower rainfall was associated with lower CD4 cell counts, unsuppressed viral loads and higher mortality. Additionally, in most quantitative studies, drought was associated with increased gaps in care and decreased ART retention in care. In qualitative studies, drought, flood, heavy rainfall and extreme heat were all linked with decreased ART uptake.

These associations should not be interpreted as suggesting direct causality between weather/climate exposures and human behaviour. Rather, the studies reviewed describe plausible indirect pathways where climate stressors exacerbate vulnerabilities, such as food insecurity, poverty and displacement, which may influence HIV-related outcomes.

Comparing findings across quantitative and qualitative studies revealed broadly consistent trends. For example, both study types identified drought as a major factor contributing to increased HIV risk behaviours due to food and financial insecurity. However, certain exposures such as floods were more commonly studied in qualitative research, potentially due to their observable immediate impact, while quantitative studies largely focused on longer term variables like drought or rainfall anomalies. These differences suggest that future research could benefit from integrating both perspectives more systematically.

While the majority of the studies on the topic are positioned within SSA and are presented in this review, there is also some evidence of the existing relationship from other regions of the world: a study in India found that drought was associated with increased sexual intimate partner violence in women.^40^ Studies on extreme weather events and HIV in the USA primarily focused on the hurricanes as the exposure: one study found that hurricanes were associated with decreased HIV testing,^41^ while the other showed no spatial correlation between major hurricanes and HIV.^42^ Additionally, in another study, wildfires negatively affected access to healthcare and mental health in PLHIV.^43^

Other health outcomes, which are primarily affected by extreme weather/climate through indirect pathways, also follow comparable trends and face related challenges: a scoping review found that extreme weather events, such as floods and droughts, were associated with adverse mental health disorders. Moreover, the groups most vulnerable to these negative effects were women, younger individuals and those working within the agriculture sector.^44^ As with our review, analyses involving longitudinal data were lacking. In another study,^28^ heavy rainfall was associated with testing positive for sexually transmitted infections (STIs) in the past 12 months. A similar finding was reported by Treibich *et al*,^29^ who showed that currently experiencing a drought significantly increases the likelihood of suffering from an STI symptom in the last 12 months, especially for women who work in the agriculture sector.

### Limitations and biases of the included studies

First, some included studies confounded weather, climate and climate change: authors often analysed temperature and rainfall patterns over a few decades and defined that as climate change, although climate is typically regarded as spanning 30 years.^45^ Attribution of particular weather to climate change is generally now possible but is usually expressed as a probability (eg, a percentage chance that climate change led to a threshold being exceeded) or as a quantitative change (eg, a number of degrees different due to climate change), rather than being able to state that climate change caused the weather. Nonetheless, many studies regarded extreme weather events as directly brought about by climate change, even though any specific weather parameter cannot always be attributed (or fully attributed) to climate change.^1^ Thus, there is a critical need for HIV researchers to collaborate directly with climate and weather scientists.

Second, only a few studies provided complete descriptions of the environmental data and the study populations: large variation in reporting led to difficulties with extracting and summarising the data. It is recommended to provide at least a basic description of the characteristics of the population studied. Nearly half of the quantitative studies were ecological. The major disadvantage of this kind of study is that conclusions about individual participants or individual-level confounders cannot be drawn from population-level data.^46^

Weather/climate exposures were not defined in a uniform way and rarely followed standardised definitions and indexes, such as Standard Precipitation Index or Standard Precipitation and Evaporation Index. Standardisation of indicators would improve comparability between studies and forming relevant conclusions and recommendations. To increase the likelihood of reporting the results in a common way, studies should present as many different weather/climate metrics as possible, such as absolute magnitude of change and percentiles.

Finally, reporting bias might also be present as some of the HIV measures, particularly sexual risk behaviours, are based on non-objective, self-reported measures. Self-reported sexual behaviour data are vulnerable to desirability bias, recall bias, lack of awareness of correct condom use and poor comprehension of survey questions. Although self-reporting remains the only existing way to investigate sexual behaviours, which might increase the risk of HIV transmission, techniques to improve recall and using a self-completed assessment might improve the validity of the data.

### Limitations of the review

While it is the first systematic review looking specifically at weather/climate and HIV in SSA, there are a number of limitations, such as publication bias. The studies showing a directional association between weather/climate exposures and HIV might have been more likely to be published than studies not finding any association. The commonality of studies investigating the relationships between droughts and HIV might reflect a publication bias, rather than the fact that droughts are more significant than other weather/climate events. Additionally, it might be possible that, due to being a slow-onset and often prolonged hazard, droughts are more likely to be associated with HIV (characterised by slow disease progression), compared with more abrupt extreme weather events, such as cyclones. Finally, relevant papers might not have been included in the review if those papers did not use terms captured by the search strategy. More broadly, however, the most significant limitation is the fragmented state of knowledge in this area. The number of eligible studies was small, with a wide variation in design, populations, exposures and outcomes. Many pathways remain poorly defined or understudied. This reflects the early stage of research on climate-related HIV vulnerabilities, highlighting the importance of continued investigation.

### Research gaps and future opportunities

The review indicates that droughts, abnormally heavy and low rainfall, floods and heat are currently associated with worsening of HIV measures. However, the access to ART has been expanding in SSA and rates of viral load suppression among people on treatment in Eastern and Southern Africa reached 93% in 2022.^47^ Additionally, conducting research on a greater variety of weather/climate and HIV associations and identifying groups most vulnerable to the negative associations between extreme weather/climate and HIV outcomes can be seen as an opportunity to reduce HIV burden. Targeting intermediate variables on weather/climate-HIV axis, such as lack of clean water and food insecurity, which have been shown to be linked with extreme weather/climate and progression in qualitative research, can also be used as a means to decrease HIV transmission and progression rates. Water and food security should be supported irrespective of weather/climate conditions, HIV and the associations, but the associations offer another (and potentially strong) impetus for addressing the insecurities. Research gaps presented below can be tackled by future research.

First, research on Western and Central Africa was lacking. Although the prevalence of HIV in Western and Central Africa is not as high as in Eastern and Southern Africa, ART coverage is much lower.^48^ Weather/climate patterns and predicted changes may differ by region: for example, by 2050, Western and Central Africa may experience a decline in mean annual rainfall of 4%–5%, while annual rainfall is predicted to increase in Eastern Africa.^49^

Moreover, all but one study^20^ focused on only one weather/climate variable in the analysis. Weather/climate variables might work synergistically to make the association with HIV measures stronger or weaker.^50^ Additionally, cumulative effects of multiple weather/climate events on HIV (eg, over many seasons) have not been investigated yet. A study on the associations between drought and migration patterns in Ethiopia, Malawi, Niger, Nigeria and Uganda found that although a single drought had a moderate effect on the probability of leaving the household, a series of droughts had a significantly higher effect.^51^ As migration is a potential contributor to higher HIV spread, as shown in qualitative studies, cumulative weather/climate events might affect HIV to a greater extent than a single event.

Furthermore, only moderation studies investigated the associations between weather/climate variables and HIV outcomes in children, and it is crucial to perform main effect analyses on this age group due to potentially different pathways of the weather/climate-HIV relationships, such as mother-to-child transmission and mixed feeding.^52^ None of the studies focused specifically on key populations, such as men who have sex with men, people who inject drugs, sex workers, transgender persons or people in prisons. It is important to investigate the impact of extreme weather/climate in these groups, as new HIV infections among key populations represented 25% of the total new infections in SSA in 2022.^53^

HIV incidence was also understudied. Most studies included in the review relied on cross-sectional study designs, therefore, directionality and temporality were difficult to establish. More longitudinal HIV datasets are needed, which contain geographical coordinates data for linkage with weather/climate variables. Longitudinal studies can be utilised for mediation analyses, which will allow identification of intermediate variables on the weather/climate-HIV axis.

The observed associations between weather/climate variables and HIV-related outcomes do not affect all populations equally and can exacerbate pre-existing health and social inequities, particularly for women, rural populations and young people. As highlighted by Ford *et al* and Wilson *et al*,^54 55^ climate justice is linked to HIV justice and failing to integrate equity into response efforts risks reinforcing systemic barriers to prevention and treatment care. Future research and interventions should be explicitly equity-focused, targeting the intersectional vulnerabilities that shape HIV risk and care disruptions during climate-related events. Even though the evidence on the topic is still scarce, the results so far already point towards potential strategies: for example, multimonth dispensing of antiretrovirals, prioritised HIV testing for vulnerable groups and food vouchers following an extreme weather event could be considered.^54^

## Conclusions

Despite possible associations found between weather/climate variability and HIV measures, primarily between droughts and HIV prevalence, there is limited research on the topic. Research gaps identified in the review can be further utilised to gain more evidence and capture the complex pathways between the studied phenomena. Further research is needed on a wider range of weather/climate variables and HIV measures and more longitudinal datasets should be collected and utilised to assess directionality and mediators of weather/climate-HIV relationships. Variables should be reported in a standardised, detailed way. Particular gaps remain in the data on Western and Central Africa, children and key populations that should be incorporated more in the future research.

## Supplementary material

10.1136/bmjph-2024-001805 online supplemental table 1

## Data Availability

Data sharing not applicable as no datasets generated and/or analysed for this study.
