# Supplementary material for: Exploring the associations of weather and climate with HIV in sub-Saharan Africa: a systematic review
Source: BMJ Public Health. 2025 Aug 18;3(2):e001805. doi: 10.1136/bmjph-2024-001805 (PMC12366556; doi:10.1136/bmjph-2024-001805)
Supplement: online supplemental table 1 [file bmjph-3-2-s001.docx]

**SUPPLEMENTARY FILES**

**Supplementary Table 1**. PRISMA checklist.

| **Section and Topic** | **Item #** | **Checklist item** | **Location where item is reported** |
| --- | --- | --- | --- |
| **TITLE** | | |  |
| Title | 1 | Identify the report as a systematic review. | Page 1 |
| **ABSTRACT** | | |  |
| Abstract | 2 | See the PRISMA 2020 for Abstracts checklist. | Page 2 |
| **INTRODUCTION** | | |  |
| Rationale | 3 | Describe the rationale for the review in the context of existing knowledge. | Page 4 |
| Objectives | 4 | Provide an explicit statement of the objective(s) or question(s) the review addresses. | Page 4 |
| **METHODS** | | |  |
| Eligibility criteria | 5 | Specify the inclusion and exclusion criteria for the review and how studies were grouped for the syntheses. | Page 6 |
| Information sources | 6 | Specify all databases, registers, websites, organisations, reference lists and other sources searched or consulted to identify studies. Specify the date when each source was last searched or consulted. | Page 6 |
| Search strategy | 7 | Present the full search strategies for all databases, registers and websites, including any filters and limits used. | Supplementary Table 2. |
| Selection process | 8 | Specify the methods used to decide whether a study met the inclusion criteria of the review, including how many reviewers screened each record and each report retrieved, whether they worked independently, and if applicable, details of automation tools used in the process. | Page 7 |
| Data collection process | 9 | Specify the methods used to collect data from reports, including how many reviewers collected data from each report, whether they worked independently, any processes for obtaining or confirming data from study investigators, and if applicable, details of automation tools used in the process. | Page 7 |
| Data items | 10a | List and define all outcomes for which data were sought. Specify whether all results that were compatible with each outcome domain in each study were sought (e.g. for all measures, time points, analyses), and if not, the methods used to decide which results to collect. | Page 5 |
|  | 10b | List and define all other variables for which data were sought (e.g. participant and intervention characteristics, funding sources). Describe any assumptions made about any missing or unclear information. | Page 5 |
| Study risk of bias assessment | 11 | Specify the methods used to assess risk of bias in the included studies, including details of the tool(s) used, how many reviewers assessed each study and whether they worked independently, and if applicable, details of automation tools used in the process. | Page 7 |
| Effect measures | 12 | Specify for each outcome the effect measure(s) (e.g. risk ratio, mean difference) used in the synthesis or presentation of results. | Page 7 |
| Synthesis methods | 13a | Describe the processes used to decide which studies were eligible for each synthesis (e.g. tabulating the study intervention characteristics and comparing against the planned groups for each synthesis (item #5)). | Page 7 |
|  | 13b | Describe any methods required to prepare the data for presentation or synthesis, such as handling of missing summary statistics, or data conversions. | Page 7 |
|  | 13c | Describe any methods used to tabulate or visually display results of individual studies and syntheses. | Page 7 |
|  | 13d | Describe any methods used to synthesize results and provide a rationale for the choice(s). If meta-analysis was performed, describe the model(s), method(s) to identify the presence and extent of statistical heterogeneity, and software package(s) used. | Page 7 |
|  | 13e | Describe any methods used to explore possible causes of heterogeneity among study results (e.g. subgroup analysis, meta-regression). | N/A |
|  | 13f | Describe any sensitivity analyses conducted to assess robustness of the synthesized results. | N/A |
| Reporting bias assessment | 14 | Describe any methods used to assess risk of bias due to missing results in a synthesis (arising from reporting biases). | Page 7 |
| Certainty assessment | 15 | Describe any methods used to assess certainty (or confidence) in the body of evidence for an outcome. | Page 7 |
| **RESULTS** | | |  |
| Study selection | 16a | Describe the results of the search and selection process, from the number of records identified in the search to the number of studies included in the review, ideally using a flow diagram. | Page 8 |
|  | 16b | Cite studies that might appear to meet the inclusion criteria, but which were excluded, and explain why they were excluded. | Figure 1. |
| Study characteristics | 17 | Cite each included study and present its characteristics. | Supplementary Table 3. and Supplementary Table 5. |
| Risk of bias in studies | 18 | Present assessments of risk of bias for each included study. | Supplementary Table 4. and Supplementary Table 7. |
| Results of individual studies | 19 | For all outcomes, present, for each study: (a) summary statistics for each group (where appropriate) and (b) an effect estimate and its precision (e.g. confidence/credible interval), ideally using structured tables or plots. | Table 2. and Supplementary Table 6. |
| Results of syntheses | 20a | For each synthesis, briefly summarise the characteristics and risk of bias among contributing studies. | Pages 8,9,11,12 |
|  | 20b | Present results of all statistical syntheses conducted. If meta-analysis was done, present for each the summary estimate and its precision (e.g. confidence/credible interval) and measures of statistical heterogeneity. If comparing groups, describe the direction of the effect. | N/A |
|  | 20c | Present results of all investigations of possible causes of heterogeneity among study results. | N/A |
|  | 20d | Present results of all sensitivity analyses conducted to assess the robustness of the synthesized results. | N/A |
| Reporting biases | 21 | Present assessments of risk of bias due to missing results (arising from reporting biases) for each synthesis assessed. | Supplementary Table 4. and Supplementary Table 7. |
| Certainty of evidence | 22 | Present assessments of certainty (or confidence) in the body of evidence for each outcome assessed. | Supplementary Table 4. and Supplementary Table 7. |
| **DISCUSSION** | | |  |
| Discussion | 23a | Provide a general interpretation of the results in the context of other evidence. | Pages 13,14 |
|  | 23b | Discuss any limitations of the evidence included in the review. | Page 14,15 |
|  | 23c | Discuss any limitations of the review processes used. | Page 15 |
|  | 23d | Discuss implications of the results for practice, policy, and future research. | Page 15 |
| **OTHER INFORMATION** | | |  |
| Registration and protocol | 24a | Provide registration information for the review, including register name and registration number, or state that the review was not registered. | N/A |
|  | 24b | Indicate where the review protocol can be accessed, or state that a protocol was not prepared. | N/A |
|  | 24c | Describe and explain any amendments to information provided at registration or in the protocol. | N/A |
| Support | 25 | Describe sources of financial or non-financial support for the review, and the role of the funders or sponsors in the review. | Page 19 |
| Competing interests | 26 | Declare any competing interests of review authors. | Page 19 |
| Availability of data, code and other materials | 27 | Report which of the following are publicly available and where they can be found: template data collection forms; data extracted from included studies; data used for all analyses; analytic code; any other materials used in the review. | Page 19 |

**Supplementary Table 2.** Search terms utilised in each literature database for the paper screening.

| **Database** | **Search terms** |
| --- | --- |
| EMBASE | (("climat*" OR "global warming" OR "weather" OR "extreme event*" OR "disaster*" OR "meteorolog*" OR "season*" OR "temperature" OR "heat*" OR "warm*" OR "hot" OR "cold" OR "frost*" OR "freez*" OR "flood*" OR "overflow*" OR "precipit*" OR "rain*" OR "landslide*" OR "slide*" OR "landslip*" OR "rockfall*" OR "mudslide*" OR "drought*" OR "aridity" OR "dry*" OR "humid*" OR "fire*" OR "wildfire*" OR "bushfire*" OR "grassfire*" OR "grass fire*" OR "woodfire*" OR "wildland fire*" OR "forest fire*" OR "woodland fire*" OR "veldt fire*" OR "moor fire*" OR "tundra fire*" OR "vegetation fire*" OR "lahar*" OR "wind speed*" OR "wind direction*" OR "cyclone*" OR "hurricane*" OR "typhoon*" OR "tornado*" OR "storm*" OR "snow*" OR "glacier*" OR "ice cap*" OR "permafrost*" OR "erosion*" OR "erod*" OR "hail*" OR "sleet*" OR "avalanche*" OR "sea level*" OR "water level*" OR "ocean acidi*" OR "ocean salinity*" OR "dissolved oxygen" OR "oxygen content*").mp. OR (exp “climate”/ OR “climate change”/ OR exp “weather”/ OR exp “extreme weather”/ OR exp “global warming”/ OR exp “temperature”/ OR exp “snow”/ OR exp “sea levels”/ OR exp “natural disasters”/ OR exp “seasons”/ OR exp “extreme heat”/ OR exp “hot temperature”/ OR exp “rain”/)) AND (("sub-Saharan Africa" OR "Africa" OR "SSA" OR "Angola" OR "Benin" OR "Botswana" OR "Burkina Faso" OR "Burundi" OR "Cameroon" OR "Cape Verde" OR "Central African Republic" OR "Chad" OR "Comoros" OR "Congo" OR "Cote d'Ivoire" OR "Djibouti" OR "Equatorial Guinea" OR "Eritrea" OR "Ethiopia" OR "Gabon" OR "Gambia" OR "Ghana" OR "Guinea" OR "Guinea-Bissau" OR "Kenya" OR "Liberia" OR "Lesotho" OR "Madagascar" OR "Malawi" OR "Mali" OR "Mauritania" OR "Mauritius" OR "Mozambique" OR "Namibia" OR "Niger*" OR "Rwanda" OR "Sao Tome and Principe" OR "Senegal" OR "Seychelles" OR "Sierra Leone" OR "Somalia" OR "Sudan" OR "Swaziland" OR "Tanzania" OR "Togo*" OR "Uganda" OR "Zambia" OR "Zimbabwe" OR "Abyssinia" OR "Basutoland" OR "Bechuanaland" OR "Benadir" OR "Biafra" OR "Bophuthatswana" OR "Bourbon Island" OR "Cape Colony" OR "Central African Empire" OR "Ciskei" OR "Dahomey" OR "Somaliland" OR "Gold Coast" OR "Hausaland" OR "Kaffraria" OR "Katanga" OR "Malagasy Republic" OR "Nyasaland" OR "Rhodesia" OR "Ruanda" OR "Songhay" OR "Spanish Guinea" OR "Tanganyika Territory" OR "Ubangi-Shari" OR "Upper Volta" OR "Zaire" OR "Zanzibar" ).mp. OR exp “Africa South of the Sahara”/) AND (("AIDS" or "Acquired Immunodeficiency Syndrome" or "Acquired Immune Deficiency Syndrome" or "HIV" or "Human Immunodeficiency Virus").mp. OR (exp "HIV"/ or exp "Acquired Immunodeficiency Syndrome"/)) |
| Global Health | (("climat*" OR "global warming" OR "weather" OR "extreme event*" OR "disaster*" OR "meteorolog*" OR "season*" OR "temperature" OR "heat*" OR "warm*" OR "hot" OR "cold" OR "frost*" OR "freez*" OR "flood*" OR "overflow*" OR "precipit*" OR "rain*" OR "landslide*" OR "slide*" OR "landslip*" OR "rockfall*" OR "mudslide*" OR "drought*" OR "aridity" OR "dry*" OR "humid*" OR "fire*" OR "wildfire*" OR "bushfire*" OR "grassfire*" OR "grass fire*" OR "woodfire*" OR "wildland fire*" OR "forest fire*" OR "woodland fire*" OR "veldt fire*" OR "moor fire*" OR "tundra fire*" OR "vegetation fire*" OR "lahar*" OR "wind speed*" OR "wind direction*" OR "cyclone*" OR "hurricane*" OR "typhoon*" OR "tornado*" OR "storm*" OR "snow*" OR "glacier*" OR "ice cap*" OR "permafrost*" OR "erosion*" OR "erod*" OR "hail*" OR "sleet*" OR "avalanche*" OR "sea level*" OR "water level*" OR "ocean acidi*" OR "ocean salinity*" OR "dissolved oxygen" OR "oxygen content*").mp. OR (exp “climate”/ OR “climate change”/ OR exp “weather”/ OR exp “global warming”/ OR exp “temperature”/ OR exp “snow”/ OR exp “natural disasters”/ OR exp “seasons”/ OR exp “rain”/)) AND (("sub-Saharan Africa" OR "Africa" OR "SSA" OR "Angola" OR "Benin" OR "Botswana" OR "Burkina Faso" OR "Burundi" OR "Cameroon" OR "Cape Verde" OR "Central African Republic" OR "Chad" OR "Comoros" OR "Congo" OR "Cote d'Ivoire" OR "Djibouti" OR "Equatorial Guinea" OR "Eritrea" OR "Ethiopia" OR "Gabon" OR "Gambia" OR "Ghana" OR "Guinea" OR "Guinea-Bissau" OR "Kenya" OR "Liberia" OR "Lesotho" OR "Madagascar" OR "Malawi" OR "Mali" OR "Mauritania" OR "Mauritius" OR "Mozambique" OR "Namibia" OR "Niger*" OR "Rwanda" OR "Sao Tome and Principe" OR "Senegal" OR "Seychelles" OR "Sierra Leone" OR "Somalia" OR "Sudan" OR "Swaziland" OR "Tanzania" OR "Togo*" OR "Uganda" OR "Zambia" OR "Zimbabwe" OR "Abyssinia" OR "Basutoland" OR "Bechuanaland" OR "Benadir" OR "Biafra" OR "Bophuthatswana" OR "Bourbon Island" OR "Cape Colony" OR "Central African Empire" OR "Ciskei" OR "Dahomey" OR "Somaliland" OR "Gold Coast" OR "Hausaland" OR "Kaffraria" OR "Katanga" OR "Malagasy Republic" OR "Nyasaland" OR "Rhodesia" OR "Ruanda" OR "Songhay" OR "Spanish Guinea" OR "Tanganyika Territory" OR "Ubangi-Shari" OR "Upper Volta" OR "Zaire" OR "Zanzibar" ).mp. OR exp “Africa South of the Sahara”/) AND (("AIDS" or "Acquired Immunodeficiency Syndrome" or "Acquired Immune Deficiency Syndrome" or "HIV" or "Human Immunodeficiency Virus").mp. OR (exp "HIV"/)) |
| PubMed | ("climat*" OR "global warming" OR "weather" OR "extreme event*" OR "disaster*" OR "meteorolog*" OR "season*" OR "temperature" OR "heat*" OR "warm*" OR "hot" OR "cold" OR "frost*" OR "freez*" OR "flood*" OR "overflow*" OR "precipit*" OR "rain*" OR "landslide*" OR "slide*" OR "landslip*" OR "rockfall*" OR "mudslide*" OR "drought*" OR "aridity" OR "dry" OR "humid*" OR "fire*" OR "wildfire*" OR "bushfire*" OR "grassfire*" OR "grass fire*" OR "woodfire*" OR "wildland fire*" OR "forest fire*" OR "woodland fire*" OR "veldt fire*" OR "moor fire*" OR "tundra fire*" OR "vegetation fire*" OR "lahar*" OR "wind speed*" OR "wind direction*" OR "cyclone*" OR "hurricane*" OR "typhoon*" OR "tornado*" OR "storm*" OR "snow*" OR "glacier*" OR "ice cap*" OR "permafrost*" OR "erosion*" OR "erod*" OR "hail*" OR "sleet*" OR "avalanche*" OR "sea level*" OR "water level*" OR "ocean acidi*" OR "ocean salinity*" OR "dissolved oxygen" OR "oxygen content*" OR “climate”[MESH] OR “climate change”[MESH] OR “weather”[MESH] OR “extreme weather”[MESH] OR “global warming”[MESH] OR “temperature”[MESH] OR “now”[MESH] OR “sea levels”[MESH] OR “natural disasters”[MESH] OR “seasons”[MESH] OR “extreme heat”[MESH] OR “hot temperature”[MESH] OR “rain”[MESH]) AND ("sub-Saharan Africa" OR "Africa" OR "SSA" OR "Angola" OR "Benin" OR "Botswana" OR "Burkina Faso" OR "Burundi" OR "Cameroon" OR "Cape Verde" OR "Central African Republic" OR "Chad" OR "Comoros" OR "Congo" OR "Cote d'Ivoire" OR "Djibouti" OR "Equatorial Guinea" OR "Eritrea" OR "Ethiopia" OR "Gabon" OR "Gambia" OR "Ghana" OR "Guinea" OR "Guinea-Bissau" OR "Kenya" OR "Liberia" OR "Lesotho" OR "Madagascar" OR "Malawi" OR "Mali" OR "Mauritania" OR "Mauritius" OR "Mozambique" OR "Namibia" OR "Niger*" OR "Rwanda" OR "Sao Tome and Principe" OR "Senegal" OR "Seychelles" OR "Sierra Leone" OR "Somalia" OR "Sudan" OR "Swaziland" OR "Tanzania" OR "Togo*" OR "Uganda" OR "Zambia" OR "Zimbabwe" OR "Abyssinia" OR "Basutoland" OR "Bechuanaland" OR "Benadir" OR "Biafra" OR "Bophuthatswana" OR "Bourbon Island" OR "Cape Colony" OR "Central African Empire" OR "Ciskei" OR "Dahomey" OR "Somaliland" OR "Gold Coast" OR "Hausaland" OR "Kaffraria" OR "Katanga" OR "Malagasy Republic" OR "Nyasaland" OR "Rhodesia" OR "Ruanda" OR "Songhay" OR "Spanish Guinea" OR "Tanganyika Territory" OR "Ubangi-Shari" OR "Upper Volta" OR "Zaire" OR "Zanzibar" OR “Africa South of the Sahara”[MESH]) AND ("AIDS" OR "Acquired Immunodeficiency Syndrome" OR “Acquired Immune Deficiency Syndrome” OR "HIV" OR "Human Immunodeficiency Virus" OR “HIV”[MESH] OR “Acquired Immunodeficiency Syndrome”[MESH]) |
| SCOPUS | TITLE-ABS-KEY (("climat*" OR "global warming" OR "weather" OR "extreme event*" OR "disaster*" OR "meteorolog*" OR "season*" OR "temperature" OR "heat*" OR "warm*" OR "hot" OR "cold" OR "frost*" OR "freez*" OR "flood*" OR "overflow*" OR "precipit*" OR "rain*" OR "landslide*" OR "slide*" OR "landslip*" OR "rockfall*" OR "mudslide*" OR "drought*" OR "aridity" OR "dry*" OR "humid*" OR "fire*" OR "wildfire*" OR "bushfire*" OR "grassfire*" OR "grass fire*" OR "woodfire*" OR "wildland fire*" OR "forest fire*" OR "woodland fire*" OR "veldt fire*" OR "moor fire*" OR "tundra fire*" OR "vegetation fire*" OR "lahar*" OR "wind speed*" OR "wind direction*" OR "cyclone*" OR "hurricane*" OR "typhoon*" OR "tornado*" OR "storm*" OR "snow*" OR "glacier*" OR "ice cap*" OR "permafrost*" OR "erosion*" OR "erod*" OR "hail*" OR "sleet*" OR "avalanche*" OR "sea level*" OR "water level*" OR "ocean acidi*" OR "ocean salinity" OR "dissolved oxygen" OR "oxygen content*") AND ("sub-Saharan Africa" OR "Africa" OR "SSA" OR "Angola" OR "Benin" OR "Botswana" OR "Burkina Faso" OR "Burundi" OR "Cameroon" OR "Cape Verde" OR "Central African Republic" OR "Chad" OR "Comoros" OR "Congo" OR "Cote d'Ivoire" OR "Djibouti" OR "Equatorial Guinea" OR "Eritrea" OR "Ethiopia" OR "Gabon" OR "Gambia" OR "Ghana" OR "Guinea" OR "Guinea-Bissau" OR "Kenya" OR "Liberia" OR "Lesotho" OR "Madagascar" OR "Malawi" OR "Mali" OR "Mauritania" OR "Mauritius" OR "Mozambique" OR "Namibia" OR "Niger*" OR "Rwanda" OR "Sao Tome and Principe" OR "Senegal" OR "Seychelles" OR "Sierra Leone" OR "Somalia" OR "Sudan" OR "Swaziland" OR "Tanzania" OR "Togo*" OR "Uganda" OR "Zambia" OR "Zimbabwe" OR "Abyssinia" OR "Basutoland" OR "Bechuanaland" OR "Benadir" OR "Biafra" OR "Bophuthatswana" OR "Bourbon Island" OR "Cape Colony" OR "Central African Empire" OR "Ciskei" OR "Dahomey" OR "Somaliland" OR "Gold Coast" OR "Hausaland" OR "Kaffraria" OR "Katanga" OR "Malagasy Republic" OR "Nyasaland" OR "Rhodesia" OR "Ruanda" OR "Songhay" OR "Spanish Guinea" OR "Tanganyika Territory" OR "Ubangi-Shari" OR "Upper Volta" OR "Zaire" OR "Zanzibar") AND ("AIDS" OR "Acquired Immunodeficiency Syndrome" OR “Acquired Immune Deficiency Syndrome” OR "HIV" OR "Human Immunodeficiency Virus" ) ) |
| Web  of Science | TS=(("climat*" OR "global warming" OR "weather" OR "extreme event*" OR "disaster*" OR "meteorolog*" OR "season*" OR "temperature" OR "heat*" OR "warm*" OR "hot" OR "cold" OR "frost*" OR "freez*" OR "flood*" OR "overflow*" OR "precipit*" OR "rain*" OR "landslide*" OR "slide*" OR "landslip*" OR "rockfall*" OR "mudslide*" OR "drought*" OR "aridity" OR "dry*" OR "humid*" OR "fire*" OR "wildfire*" OR "bushfire*" OR "grassfire*" OR "grass fire*" OR "woodfire*" OR "wildland fire*" OR "forest fire*" OR "woodland fire*" OR "veldt fire*" OR "moor fire*" OR "tundra fire*" OR "vegetation fire*" OR "lahar*" OR "wind speed*" OR "wind direction*" OR "cyclone*" OR "hurricane*" OR "typhoon*" OR "tornado*" OR "storm*" OR "snow*" OR "glacier*" OR "ice cap*" OR "permafrost*" OR "erosion*" OR "erod*" OR "hail*" OR "sleet*" OR "avalanche*" OR "sea level*" OR "water level*" OR "ocean acidi*" OR "ocean salinity*" OR "dissolved oxygen" OR "oxygen content*") AND ("sub-Saharan Africa" OR "Africa" OR "SSA" OR "Angola" OR "Benin" OR "Botswana" OR "Burkina Faso" OR "Burundi" OR "Cameroon" OR "Cape Verde" OR "Central African Republic" OR "Chad" OR "Comoros" OR "Congo" OR "Cote d'Ivoire" OR "Djibouti" OR "Equatorial Guinea" OR "Eritrea" OR "Ethiopia" OR "Gabon" OR "Gambia" OR "Ghana" OR "Guinea" OR "Guinea-Bissau" OR "Kenya" OR "Liberia" OR "Lesotho" OR "Madagascar" OR "Malawi" OR "Mali" OR "Mauritania" OR "Mauritius" OR "Mozambique" OR "Namibia" OR "Niger*" OR "Rwanda" OR "Sao Tome and Principe" OR "Senegal" OR "Seychelles" OR "Sierra Leone" OR "Somalia" OR "Sudan" OR "Swaziland" OR "Tanzania" OR "Togo*" OR "Uganda" OR "Zambia" OR "Zimbabwe" OR "Abyssinia" OR "Basutoland" OR "Bechuanaland" OR "Benadir" OR "Biafra" OR "Bophuthatswana" OR "Bourbon Island" OR "Cape Colony" OR "Central African Empire" OR "Ciskei" OR "Dahomey" OR "Somaliland" OR "Gold Coast" OR "Hausaland" OR "Kaffraria" OR "Katanga" OR "Malagasy Republic" OR "Nyasaland" OR "Rhodesia" OR "Ruanda" OR "Songhay" OR "Spanish Guinea" OR "Tanganyika Territory" OR "Ubangi-Shari" OR "Upper Volta" OR "Zaire" OR "Zanzibar") AND ("AIDS" OR "Acquired Immunodeficiency Syndrome" OR “Acquired Immune Deficiency Syndrome” OR "HIV" OR "Human Immunodeficiency Virus")) |

**Supplementary Table 3.** Characteristics of quantitative studies included in the review.

| **Authors**  **(year)** | **Study aims** | **Study design** | **Sample characteristics** | **Countries** | **Type of weather/ climate variable(s)** | **Type of HIV measure(s)** | **Key conclusions** |
| --- | --- | --- | --- | --- | --- | --- | --- |
| Austin et al (2021) | To understand the underlying role of environmental change and disasters in conditioning vulnerabilities to HIV in women. | Ecological | Females living with HIV^1^ aged ≥15 years old | Eswatini, Ethiopia, Niger, Angola, Gambia, The Nigeria, Ghana, Benin, Guinea, Guinea-Bissau, Rwanda, Senegal, Botswana, Burkina Faso, Sierra Leone, Burundi, Somalia, Cabo Verde, South Africa, Kenya, South Sudan, Cameroon, Central African Republic, Sudan, Chad, Lesotho, Liberia, Comoros, Madagascar, Tanzania, Congo, Democratic Republic, Malawi, Togo, Congo, Rep, Mali, Uganda, Cote d’Ivoire, Mauritania, Mauritius, Djibouti, Zambia, Mozambique, Zimbabwe, Eritrea, Namibia | Drought | HIV prevalence, condomless sex | Less-developed countries with elevated suffering from droughts have increased levels of moderate and severe food insecurity, which increases the percentage of HIV cases among women. |
| Baker  (2020) | To test the effect of long-term temperature changes on HIV prevalence. | Ecological | 400,000 individuals | Burkina Faso, Burundi, Cameroon, Congo – Kinshasa, Côte d’Ivoire, Ethiopia, Gabon, Ghana, Guinea, Kenya, Lesotho, Liberia, Malawi, Mali, Mozambique, Namibia, Rwanda, Senegal, Sierra Leone, Swaziland, Tanzania, Togo, Uganda, Zambia, Zimbabwe | Temperature variation | HIV prevalence, transactional sex | Warmer periods are linked with an increase in HIV prevalence, particularly for younger generations. |
| Bakshi et al (2019) | To examine the climate-mortality relationship using a set of climate variability measures | Ecological | 850,736 households; Individuals aged 15-49 years old | Kenya, Mali, Malawi | Heat waves, cold snaps,  drought, heavy rainfall | HIV prevalence | Drought and heavy rainfall-related increases in mortality are strongest in regions with high HIV prevalence rates, but no trend is seen during heat waves and cold snaps. |
| Burke et al (2015) | To understand how economic conditions shape HIV risks. | Ecological | 203,796 individuals aged 15-49 years old | Swaziland, Lesotho, Zambia, Zimbabwe, Malawi, Mozambique, Tanzania, Kenya, Cameroon, Rwanda, Ghana, Burkina Faso, Liberia, Guinea, Sierra Leone, Ethiopia, Mali, Congo Democratic Republic, Senegal | Negative rainfall shock | HIV prevalence | Deterioration in economic conditions, in the form of rainfall-related income shocks, contributes significantly to both village and country-level rates of HIV infection in sub-Saharan Africa. |
| Epstein et al (2023) | To describe the associations between drought, HIV testing, and HIV transmission risk. | Cross-sectional | 206,205 individuals aged 15-49 years old (females) or 15-59 years old (males); 34.1% male | Cameroon, Kenya, Lesotho, Mozambique, Malawi, Namibia, South Africa, Uganda, Zambia, Zimbabwe | Drought | HIV testing, condomless sex, number of sexual partners (in the prior 12 months) | Drought was associated with lower HIV testing and higher probability of condomless sex at their last sexual encounter. |
| Iwuji et al  (2023) | To investigate the impact of drought on ART adherence and retention in HIV care. | Longitudinal | 40,714 individuals; 31.7% male | South Africa | Drought | ART^2^ medication possession ratio in the 6-monthsafter ART initiation; retention in care | There was a marked decrease in ART adherence as measured by MPR during the drought years with no full recovery of adherence during the wet years. There was also a marked drop in retention in care at the start of the drought years followed by some recovery and then, a dip again towards the end of the observation period. |
| Low et al  (2019) | To assess whether people living in areas most severely affected by the drought had higher HIV prevalence, changes in risk behaviours, and viral load suppression. | Cross-sectional | 12887 individuals aged 15-59 years old; 50.1% male | Lesotho | Drought | HIV prevalence, viral load suppression,  condom use at last sex, ART treatment, history of selling sex or forced sex, number of sexual partners | Drought was associated with higher HIV prevalence in rural girls aged 15–19. Intergenerational, transactional sex, and condomless sex contribute to increased HIV prevalence during drought. Drought was not associated with higher risk of unsuppressed viral loads nor ART. |
| Mason et al (2005) | To investigate trends in child malnutrition in relation to HIV/drought. | Ecological | Children aged 5-59 months old | Lesotho, Malawi, Mozambique, Swaziland, Zambia, Zimbabwe | Drought | HIV prevalence | The interaction between drought period and HIV prevalence was found to be highly significant. |
| Mason, Chotard et al (2010) | To assess the size of the intermittent effects of drought, food insecurity, and HIV on child malnutrition. | Ecological | Children aged 6-59 months old | Lesotho, Malawi, Mozambique, Swaziland, Zambia, Zimbabwe, Kenya, Ethiopia, Eritrea, Somalia, Sudan, Uganda | Drought | HIV prevalence | Drought and HIV are not themselves related, but they may interact with each other. |
| Mason, Jayne et al (2010) | To measure the extent to which HIV/ AIDS exacerbates the impacts of drought on agricultural production. | Ecological | Individuals living with HIV | Zambia | Drought | HIV prevalence | There was no statistically significant evidence that HIV/AIDS^3^ exacerbates the effects of drought on crop output/hectare. |
| Nagata et al (2022) | To determine the association between heavy rainfall, HIV risk behaviours and HIV prevalence. | Cross-sectional | 288,333 individuals from 21 SSA countries aged 15-59 years old; 40.2% male | Angola, Burkina Faso, Burundi, Cameroon, Chad, Congo Democratic Republic, Cote d'Ivoire, Ethiopia, Gabon, Ghana, Lesotho, Malawi, Mozambique, Rwanda, Senegal, Sierra Leone, Tanzania, Togo, Uganda, Zambia, Zimbabwe | Heavy rainfall | HIV prevalence, number of sexual partners in the past 12 months | Heavy rainfall was associated with higher HIV prevalence, particularly in rural areas, in adults aged ≥30 years and in younger adults. |
| Treibich et al (2022) | To analyse the effects of drought on risk behaviours of men and women. | Cross-sectional | 14,311 individuals aged 15-49 years old; 46.1% male | Malawi | Drought | HIV prevalence, transactional sex | Amongst women employed in agriculture, droughts doubled the likelihood of engaging in transactional sex. Furthermore, each drought increased HIV prevalence by 15%. |
| Trickey et al (2023) | To investigate whether living somewhere that has recently had low levels of rainfall is associated with adverse ART outcomes. | Longitudinal | 270,708 PLHIV on ART treatment aged ≥16 years old; 32.9% male | Lesotho, Malawi, Mozambique, South Africa, Zambia, and Zimbabwe | Rainfall variability | Mortality, CD4 cell count, HIV viral loads, >12-month gaps in follow-up | Mortality was higher among PWH on ART living in regions with lower rainfall than usual. The odds of unsuppressed viral loads among PWH on ART were higher in areas with lower rainfall than historically. |
| Trickey et al (2024) | To analyse the associations connecting drought with HIV (sexual behaviours and incidence) via poverty. | Cross-sectional | 102,081 respondents aged 15-59 years old | Eswatini, Lesotho, Tanzania, Uganda, and Zambia | Drought | HIV incidence (Recent HIV) | Women in rural areas who had recently experienced drought had increased odds of having recently acquired HIV. High-risk sex among all women and intergenerational sex among urban women also increased the odds of having recently acquired HIV. |

ART: Antiretroviral therapy, PLHIV: People living with HIV

**Supplementary Table 4.** Quality assessment of quantitative studies included in the review.

| **Study ID** | **1. Was the study design appropriate for the stated aim(s)?** | **2. Was the study population clearly specified and defined?** | **3. Were all the subjects selected from the same or similar populations?** | **4. Were inclusion and exclusion criteria for being in the study prespecified and applied uniformly to all participants?** | **5. Was the exposure accurately measured to minimise bias?** | **6. Was the outcome accurately measured to minimise bias?** | **7. Were key potential confounding variables measured and adjusted statistically?** | **8. Is missing data accounted for/minimal, so that it does not raise bias?** | **9. Were the results for the analyses described in the methods, presented?** | **10. Were the results internally consistent?** | **11. Is the study free from any technical flaws in the methodology/ interpretation?** | **Score**  **(out of 11)** |
| --- | --- | --- | --- | --- | --- | --- | --- | --- | --- | --- | --- | --- |
| Austin et al (2021) | 1 | 0 | 0 | 1 | 0 | 0 | 0 | 1 | 1 | 1 | 0 | **5** |
| Baker (2020) | 0 | 0 | 0 | 0 | 1 | 1 | 1 | 1 | 1 | 1 | 0 | **6** |
| Bakshi et al (2019) | 1 | 1 | 1 | 1 | 1 | 1 | 0 | 1 | 0 | 0 | 1 | **8** |
| Burke et al (2015) | 0 | 1 | 0 | 1 | 1 | 1 | 0 | 1 | 1 | 1 | 0 | **7** |
| Epstein et al (2023) | 1 | 1 | 0 | 1 | 1 | 0 | 1 | 0 | 1 | 1 | 1 | **8** |
| Iwuji et al (2023) | 1 | 1 | 1 | 1 | 1 | 1 | 1 | 1 | 1 | 1 | 1 | **11** |
| Low et al (2019) | 0 | 1 | 1 | 1 | 1 | 1 | 1 | 0 | 1 | 0 | 1 | **8** |
| Mason et al (2005) | 1 | 1 | 0 | 0 | 0 | 1 | 0 | 1 | 0 | 1 | 0 | **5** |
| Mason, Chotard et al (2010) | 1 | 1 | 0 | 0 | 1 | 1 | 0 | 1 | 0 | 0 | 0 | **5** |
| Mason, Jayne et al (2010) | 1 | 0 | 1 | 1 | 1 | 0 | 0 | 1 | 0 | 0 | 0 | **5** |
| Nagata et al (2022) | 1 | 1 | 0 | 1 | 1 | 1 | 1 | 1 | 1 | 0 | 1 | **9** |
| Treibich et al (2022) | 0 | 1 | 1 | 1 | 1 | 1 | 1 | 1 | 1 | 1 | 1 | **10** |
| Trickey et al (2023) | 1 | 1 | 1 | 1 | 1 | 1 | 0 | 0 | 1 | 0 | 1 | **8** |
| Trickey et al (2024) | 1 | 1 | 1 | 1 | 1 | 1 | 1 | 1 | 1 | 0 | 1 | **10** |

Yes=1 point, No=0 points

**Supplementary Table 5.** Characteristics of qualitative studies included in the review.

| **Study ID** | **Study aims** | **Study design** | **Sample characteristics** | **Country** | **Type of weather/**  **climate event** | **Type of HIV measure(s)** | **Key conclusions** |
| --- | --- | --- | --- | --- | --- | --- | --- |
| Anthonj et al (2015) | To provide a deeper understanding of the impact that flooding has on PLHIV and HIV services. | Semi-structures interviews, expert interviews, focus group discussion, feedback meeting | 7 females living with HIV affected by flooding, 9 members of civil society organisations, 7 representatives from public sector institutions | Ohangwena Region, Namibia | Flood | Forced sex, condom use, ART treatment, HIV testing | PLHIV in Ohangwena are more vulnerable in the case of flooding than people not affected by HIV. Flooding is a barrier for. accessibility of health services and ART treatment. |
| Githinji et al (2014) | To analyse the mechanisms through which HIV/AIDS and climate variability overlap. | Ethnographic research approach | 311 participants aged at least 18 years old; There were 184 participants in the initial phase, 97 participants in the second phase, and 30 female household heads in the third phase. | Nsisha, Tanzania | Drought and heavy rain | ART treatment, transactional sex | Vulnerabilities to various stresses—climate variability, food insecurity, social marginalization, HIV/AIDS—interact in ways that both create and compound negative effects on human well-being. |
| Kandawasvika et al (2021) | To document the public health impact of cyclones Idai on PLHIV. | Interviews and rapid assessment | Senior nurses at health facilities | Chimanimani, Zimbabwe | Cyclone (Idai) | ART treatment demand, access to ART treatment | Established health programmes such as HIV were disrupted in following Cyclone Idai. |
| O'Laughin et al (2021) | To explore the influence to engagement in HIV clinical care: individual, social environment, physical environment, and regulatory policies. | Semi-structured interviews | 47 individuals attending the clinic with the average age of 32 years old and 8 staff members with the average age of 33 years old; 57% of client participants were male and 75% of clinic staff participants were male | Nakivale Refugee Settlement, Uganda | Heavy rain and heat | HIV clinic attendance (ART treatment) | Extreme rain and heat patterns lowered HIV attendance due to inability to walk long distances and food insecurity which would be a priority over clinic attendance. |
| Orievulu et al (2022) | To show how the experience of the 2015 drought may have contributed to vulnerabilities on HIV care. | In-depth interviews and thematic analysis | 27 PLHIV with a median age of 39.5 years old; 44.4% of the sample was male | Hlabisa, South Africa | Drought | ART treatment | Drought exacerbated existing, and caused additional, challenges and competing priorities to PLHIV’s continuum of care. This was mediated through restricted food and water intakes, migration and the loss of identity through livestock losses. |
| Tran et al (2023) | To gain insights about the implications of interruptions in HIV care engagement and viral suppression. | In-depth interviews | 10 participants who provided care for patients at the health facilities | Busia and Trans Nzoia, Western Kenya | Flood | Interruption in HIV care (ART treatment) | Flooding limited access to ART treatment due to clinics closing, populations being displaced and food insecurity. |

ART: Antiretroviral therapy, PLHIV: People living with HIV

**Supplementary Table 6.** Numerical summary of weather/climate exposure-HIV measure association pairs used in the included qualitative studies.

|  | | **Precipitation changes** | | | **Temperature changes** | **Wind changes** |
| --- | --- | --- | --- | --- | --- | --- |
|  |  | Drought (N=2) | Heavy rainfall (N=2) | Flood (N=2) | Heat (N=1) | Cyclones (N=1) |
| **HIV risk behaviours** | Condom use (N=1) | 0 | 0 | 1 | 0 | 0 |
|  | Transactional sex (N=1) | 1 | 0 | 0 | 0 | 0 |
|  | Forced sex/sexual violence (N=1) | 0 | 0 | 1 | 0 | 0 |
| **HIV transmission measures** | HIV testing/diagnosis (N=1) | 0 | 0 | 1 | 0 | 0 |
| **HIV progression measures** | ART treatment demand/access (N=6) | 2 | 2 | 2 | 1 | 1 |
|  | No studies investigating the association | | | | | |
|  | One study investigating the association | | | | | |
|  | Two or more studies investigating the association | | | | | |

ART: Antiretroviral therapy

**Supplementary Table 7.** Quality assessment of qualitative studies included in the review.

| **Study ID** | **1. Was the study design appropriate for the stated aim(s)?** | **2. Was the study population clearly specified and defined?** | **3. Were all the subjects selected or recruited from the same or similar populations?** | **4. Were inclusion and exclusion criteria for being in the study prespecified and applied uniformly to all participants?** | **5. Is there congruity between the research methodology and the interpretation of results?** | **6. Is there a statement locating the researcher culturally or theoretically?** | **7. Are the findings adequately derived from the data?** | **Score (out of 7)** |
| --- | --- | --- | --- | --- | --- | --- | --- | --- |
| Anthonj et al (2015) | 0 | 0 | 1 | 1 | 1 | 0 | 1 | **4** |
| Githinji et al (2014) | 0 | 1 | 1 | 1 | 1 | 0 | 0 | **4** |
| Kandawasvika et al (2021) | 0 | 0 | 1 | 0 | 0 | 0 | 0 | **1** |
| O'Laughin et al (2021) | 0 | 1 | 1 | 1 | 1 | 0 | 1 | **5** |
| Orievulu et al (2022) | 1 | 1 | 1 | 1 | 1 | 0 | 1 | **6** |
| Tran et al (2023) | 1 | 0 | 1 | 0 | 1 | 0 | 1 | **4** |

Yes=1 point, No=0 points
